# Decreased activity in the reward network of chronic insomnia patients

**DOI:** 10.1038/s41598-020-79989-2

**Published:** 2021-02-11

**Authors:** Yuki Motomura, Ruri Katsunuma, Naoko Ayabe, Kentaro Oba, Yuri Terasawa, Shingo Kitamura, Yoshiya Moriguchi, Akiko Hida, Yuichi Kamei, Kazuo Mishima

**Affiliations:** 1 Department of Sleep-Wake Disorders, National Institute of Mental Health, National Center of Neurology and Psychiatry, 4-1-1 Ogawa-Higashi, Kodaira, Tokyo 187-8553 Japan; 2 Faculty of Design, Kyushu University, 4-9-1 Shiobaru, Minami-ku, Fukuoka, 815-8540 Japan; 3 Integrative Brain Imaging Center, National Center of Neurology and Psychiatry, 4-1-1 Ogawa-Higashi, Kodaira, Tokyo 187-8553 Japan; 4 Department of Regional Studies and Humanities, Faculty of Education and Human Studies, Akita University, 1-1, Tegata-Gakuenmachi, Akita, 010-8502 Japan; 5 Department of Human Brain Science, Institute of Development, Aging and Cancer, Tohoku University, 4-1 Seiryo-machi, Aoba-ku, Sendai, 980-8575 Japan; 6 Department of Psychology, Keio University, 4-1-1 Hiyoshi, Kohoku-ku, Yokohama, Kanagawa 223-8521 Japan; 7 Kamisuwa Hospital, 1-17-7 Ote, Suwa, Nagano, Japan; 8 Faculty of Medicine, Akita University, 1-1-1 Hondo, Akita, 010-8543 Japan; 9 International Institute for Integrative Sleep Medicine, University of Tsukuba, 1-1-1 Tennodai, Tsukuba, Ibaraki Japan

**Keywords:** Neuroscience, Emotion, Reward, Psychiatric disorders

## Abstract

In modern society, many people have insomnia. Chronic insomnia has been noted as a risk factor for depression. However, there are few functional imaging studies of the brain on affective functions in chronic insomnia. This study aimed to investigate brain activities induced by emotional stimuli in chronic insomnia patients. Fifteen patients with primary insomnia and 30 age and gender matched healthy controls participated in this study. Both groups were presented images of fearful, happy, and neutral expressions consciously and non-consciously while undergoing MRI to compare the activity in regions of the brain responsible for emotions. Conscious presentation of the Happy-Neutral contrast showed significantly lower activation in the right orbitofrontal cortex of patients compared to healthy controls. The Happy-Neutral contrast presented in a non-conscious manner resulted in significantly lower activation of the ventral striatum, right insula, putamen, orbitofrontal cortex and ventral tegmental area in patients compared to healthy controls. Our findings revealed that responsiveness to positive emotional stimuli were decreased in insomniac patients. Specifically, brain networks associated with rewards and processing positive emotions showed decreased responsiveness to happy emotions especially for non-conscious image. The magnitude of activity in these areas also correlated with severity of insomnia, even after controlling for depression scale scores. These findings suggest that insomnia induces an affective functional disorder through an underlying mechanism of decreased sensitivity in the regions of the brain responsible for emotions and rewards to positive emotional stimuli.

## Introduction

Several recent large-scale studies have shown that approximately one-fourth of adults complain of sleep problems, and that 10–15% of them trouble functioning during the day because of insomnia. Additionally, around 6–10% of the general population meet the clinical diagnostic criteria of chronic insomnia^1^. Insomnia has a high prevalence, and it can cause various physical and psychological symptoms such as depression, psychomotor impairment, and easy fatigability. Thus, it is a common disease that should not be overlooked in public health studies. Insomnia has been found to accompany many other psychiatric conditions, including depression, and patients with depression have experienced a decrease in symptoms after receiving treatment for insomnia^2,3^. Strong mutual relationships between sleep problems and mood modulation are thus expected.

Furthermore, chronic insomnia has been identified as a risk factor for depression. Ford et al. surveyed 7954 people in the general population and found that those with insomnia, both at baseline and at the one-year follow-up (persistent insomniacs), had a 40-fold higher risk of developing depression than healthy sleepers^4^. A more recent cohort study^5^ also showed a two-fold increase in the risk of depression 3.5 years after being diagnosed with insomnia. Furthermore, a meta-analysis reported that the odds of depression increased by 2.1 in people with insomnia^6^, which confirms that chronic insomnia is a risk factor for depression. Additionally, insomnia has been found to be a risk factor for anxiety disorders^7^.

The “hyperarousal” theory states that insomnia patients are in states of excessive alertness, both physiologically and psychologically^8–10^; this is considered as an explanation for the psychological and physiological symptoms in insomnia. An increased heart rate, increased sympathetic nerve activity in heart rate variability, decreased parasympathetic nerve activity^11–13^, higher levels of cortisol^14–18^, decreased melatonin levels^19,20^, increased noradrenaline levels^21^, increased brain glucose metabolism^22,23^, hyperarousal of the cortex (observed as rapid waves on electroencephalography)^24–29^, and many other findings supporting physiological and psychological hyperactivity are observed in individuals with difficulty sleeping, including insomnia patients. Hyperarousal has also been associated with the symptoms of depression in a cohort study^30^.

Multiple studies have investigated the personality traits associated with insomnia and mood modulation. Kales et al.^31^ conducted a survey using the Minnesota Multiphasic Personality Inventory in insomnia patients and found that they had high scores in the depression, psychasthenia, and conversion hysteria subscales, suggesting the presence of mood modulation problems in insomnia patients. Based on these results, an internalization model for handling conflicts in insomnia patients has been proposed, which is associated with the physiological hyperarousal and individual characteristics of insomnia, wherein internal conflicts induce high levels of emotional arousal^32^. In a study by Koffel and Watson^33^, 349 healthy young adults, 213 older adults, and 213 insomnia patients were surveyed to study the associations between the problems related to insomnia during the night (prolonged sleep latency, difficulty maintaining sleep) and during the day (fatigue, drowsiness), depression, and anxiety. They found that both day- and night-time problems were associated with anxiety, depression, post-traumatic stress disorder, and social phobia. However, anxiety and depression were more strongly associated with the day-time problems of insomnia. Furthermore, day-time problems were associated with a high negative emotional reactivity and low positive emotional reactivity in a survey using the Positive and Negative Affect Schedule. This suggests that day-time sleep-related symptoms are associated with a greater risk for depression, which can be caused by insomnia, than night-time symptoms.

In addition to the night-time symptoms, it is necessary to investigate the mechanisms underlying the decreased affective function during day-time arousal in chronic insomnia patients. Recent neuroimaging studies have revealed that these patients have altered functional coupling networks in the amygdala at rest when compared with that in healthy individuals^34,35^. Baglioni et al. reported increased amygdala activity levels in response to sleep-related stimuli in patients with insomnia^36^. Although they found that in healthy individuals, the levels of activity were proportional to the magnitude of arousal generated in response to common affective stimuli, there were no changes in the magnitude of the negative valence. A study by Klumpp et al.^37^ is an example of a similar attempt that has been made previously; they reported that worse sleep quality (i.e., a higher Pittsburgh sleep quality index [PSQI] total scores) predicted increased left amygdala-subgenual anterior cingulate functional connectivity and reduced its connectivity with the posterior cerebellar lobe and superior temporal gyrus. However, neurofunctional imaging studies related to affective function in chronic insomnia are rare, and the detailed mechanisms underlying the changes in activity due to insomnia are unknown. In this study, we used facial expression tasks with the aim of investigating the brain areas associated with affective states provoked by representations of facial expressions in patients with severe chronic insomnia.

## Methods

### Ethical considerations

This study was approved by the ethics committee of the National Center of Neurology and Psychiatry, and conforms to the principles of the Declaration of Helsinki. All participants provided written informed consent.

### Participants

The participants included 15 patients who visited the National Center of Neurology and Psychiatry Hospital Sleep Disorders Center and were diagnosed with primary insomnia as defined in the Diagnostic and Statistical Manual of Mental Disorders and 30 age- and sex-matched healthy individuals who were recruited for this study. The experiment was conducted between January 2011 and December 2014.

### Experimental protocol

#### Insomnia patient group

Fifteen patients were assigned to the insomnia patient (PT) group. Magnetic resonance imaging (MRI) was conducted after a 3-day washout period for hypnotics, managed by the attending physician. Participants filled out the PSQI^38^ and Athens Insomnia Scale (AIS)^39^ after arriving at the center to evaluate the severity of their sleep disorder or insomnia and the Zung Self-Rating Depression Scale (SDS)^40^ to evaluate the severity of their depression. Participants aged ≥ 60 years also underwent the Mini-Mental State Examination (MMSE)^41^ before they performed the emotional-face viewing task. The experiments were conducted between 10:00 and 15:00.

Insomnia patients were excluded if they met the following criteria: patients with serious physical condition, psychiatric diseases, sleep disorders other than primary insomnia, implanted metal devices such as pacemakers, ophthalmic conditions including abnormal color perception, patients doing shift work, patients who had traveled abroad to a region with a 6-h or greater time difference in the past 3 months, patients with 200 mg/day or more caffeine intake, heavy smokers (those who would find it stressful to abstain from smoking for 5 days), and patients suspected to have cognitive decline (MMSE score ≤ 25 points).

#### Healthy control group

The healthy control (HC) group comprised thirty healthy individuals, age- and sex-matched to the insomnia patients. They visited the study site a week before the experiment for briefings on the nature and purpose of the experiment, to provide their written consent, and to complete online questionnaires to confirm the absence of pre-existing sleep disorders. They filled out the following questionnaires to screen for certain suspected diseases: the PSQI^38^ and AIS^39^ for insomnia, Epworth Sleepiness Scale^42^ for hypersomnia, STOP-Bang questionnaire^43^ for sleep apnea, the International Restless Legs Syndrome Study Group rating scale for the severity of restless legs syndrome^44^ and parasomnia, the Munich Parasomnia Screening^45^ for sleep behavior disorder, and the Rapid Eye Movement Sleep Behavior Disorder Screening Questionnaire^46^ for rapid eye movement sleep behavior disorder.

A sleep–wake cycle survey by actigraphy and sleep apnea screening and pulse measurement using pulse oximetry were conducted for the first night at home for one week. Participants revisited the laboratory one week after the tests at home to fill the same questionnaires as the PT group, undergo the MMSE, and perform the emotional-face viewing task. The tests were conducted between 10:00 and 15:00.

The exclusion criteria for the healthy participants were as follows: those with any of the following sleep disorders—insomnia, hypersomnia, sleep apnea, parasomnia, rapid eye movement sleep behavior disorder, or circadian rhythm sleep disorder; any serious physical conditions, use of medication or other substances that may affect the results of the study (hypnotics, drugs that induce sleepiness such as anti-histamines and steroids); psychiatric diseases; implanted metal devices such as pacemakers; ophthalmic conditions including abnormal color perception; individuals doing shift work; individuals who had traveled abroad to a region with a 6-h or greater time difference in the past 3 months; individuals with 200 mg/day or more caffeine intake, heavy smokers (those who would find it stressful to abstain from smoking for 5 days); and patients with suspected cognitive decline (an MMSE score of ≤ 25).

### Emotional-face viewing task

**Figure 1:**
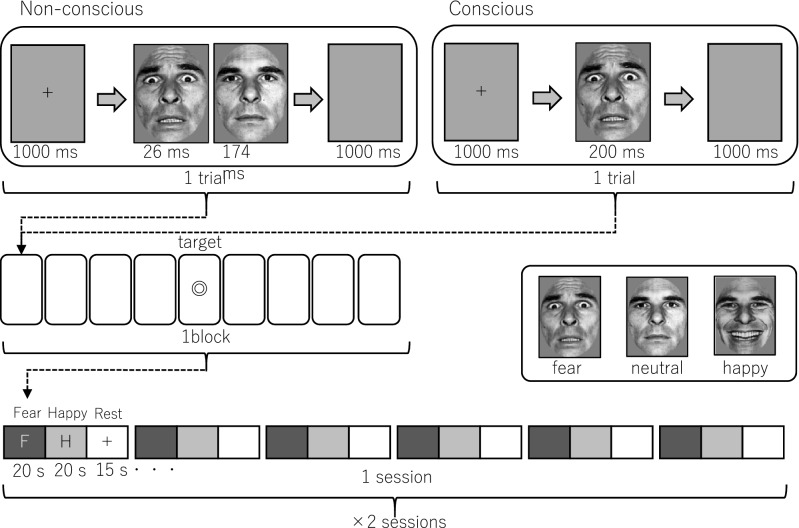
Emotional-face viewing task. Facial pictures depicting fearful, happy, or neutral expressions were used as the stimuli and were presented either non-consciously or consciously. In a non-conscious trial, an emotional image (either fearful, happy, or neutral) was implicitly presented for 26 ms, followed by an explicit presentation of a neutral “masking” face of the same individual as the preceding implicit emotional face (when the implicit face was neutral, the following explicit mask was of a different person of the same sex) for 174 ms. Participants were required to press a button in response to each “target” stimulus to keep themselves awake during the scanning.

The emotional-face viewing task developed by one of the authors of this study^47^ was conducted while the participants underwent MRI as shown in Fig. 1. The participants were presented images of emotional facial expressions under the following conditions: (1) a conscious condition wherein the emotional facial expression image was presented for long enough for the participant to visually recognize it consciously or (2) a non-conscious condition, wherein the emotional facial expression image was presented for too short a duration for the participant to consciously recognize it, following which it was masked with a neutral expression. The 48 images used as stimuli in this study were selected from two standardized facial stimuli sets (Ekman and Friesen^48^ and Advanced Telecommunications Research Institute International^49^: http://www.atr-p.com/products/face-db.html), and included fearful, happy, and neutral faces from four Japanese and four Western individuals of both genders. For both the conscious and non-conscious conditions, a point image was presented for 1000 ms to guide the participants’ gaze. Following this, for the conscious condition, one of the facial expressions (fearful, happy, or neutral) was presented for 200 ms. For the non-conscious condition, one of the facial expressions was presented for 26 ms, after which the image was backward-masked by a neutral face from the same individual. When a neutral image was presented first, an image of the neutral expression from a different individual of the same sex was presented for backward masking. Images were presented in blocks of eight trials. The target stimulus was presented randomly during each block, and participants were asked to press a button to react. During the rest block, the gazing point image was presented for 15 s. Each of the two sessions consisted of the following sequence repeated six times with different kinds of facial expressions: non-conscious condition block → conscious condition block → rest block. The second session was conducted after a 1–2-min break.

After the completion of each session, the participants were asked to report their subjective sleepiness during the task on the following scale: 0 = Not sleepy at all, 1 = Slightly sleepy, 2 = Sleepy, 3 = Very sleepy, and 4 = I fell asleep for a little bit. The mean scores of the subjective sleepiness for the two sessions were calculated for analysis.

After completing the two sessions, the participants were asked to report how they saw the masked face using the following scale: 0 = It was completely unrecognizable, 2 = I saw something, but I was not sure what kind of expression it was, 3 = I was able to recognize the expression in some of the pictures, and 4 = I recognized the expression in almost all the pictures.

There were no participants who answered 3 or 4 with regards to how they saw the masked face.

### Functional MRI data acquisition

A 3 T MRI Verio (Siemens AG. München, Germany) was used for the functional MRI (fMRI). Single-shot echo-planar imaging [TR/TE = 2500 ms/25 ms, 30 axial slices, voxel size = 6 mm × 6 mm × 4 mm, 1 mm inter-slice gap, flip angle 90°, matrix size = 64 × 64, field of view = 384 mm × 384 mm] was used for the functional imaging of the participants while they executed the task. During each session, 137 scans were taken, and the first five scans were discarded from analysis.

### fMRI data analysis

The SPM8 (Wellcome Department of Imaging Neuroscience; http://www.fil.ion.ucl.ac.uk/spm/software/spm8/) was used for the brain functional imaging data analyses. Functional images were motion and slice timing-corrected, co-registered to a magnetization prepared-rapid gradient echo structural image, spatially normalized to the Montreal Neurological Institute template, and smoothed with an 8 mm full-width half maximum Gaussian kernel. The time-series data including the 3D blood-oxygen-level-dependent signals of the participants were analyzed with the general linear model (first-level fixed model effect). The time-series of each session was convolved with the canonical hemodynamic response implemented function in SPM8, and the functions of the hypothesized hemodynamics for the various conditions of the stimulus presentation were created. The time-series of the blood flow models for the responses to the six emotional and conscious stimulus categories (non-conscious/conscious happy, non-conscious/conscious fear, and non-conscious/conscious neutral) and the time-series data of the variables related to the six body movements were built into the design matrix as a regressor. Actual blood-oxygen-level-dependent signals were analyzed voxel by voxel with a general linear model, and the beta values corresponding to each regressor were calculated.

For inter-participant testing, the beta values between the conditions of interest for each voxel were analyzed using a paired t-test. The contrast (activity from viewing the neutral expression image subtracted from the activity from viewing the emotional expression image) was then created. A total of four contrasts for the fear–neutral and happy–neutral expressions presented consciously and non-consciously were generated.

Areas known to have important associations with positive or negative emotions, i.e., the amygdala, insula, anterior cingulate cortex, and areas involved in responding to pleasurable stimuli or motivation for reward^50–57^, were selected as regions of interest. Masks for the right and left amygdala, right and left insula, anterior cingulate cortex, corpus striatum (putamen + caudate nucleus), and right and left orbitofrontal areas were created using the Anatomical Automatic Labeling from the SPM WFU PickAtlas toolbox (Wake Forest University; http://fmri.wfubmc.edu/downloads/WFU_PickAtlas_User_Manual.pdf). A mask for the ventral tegmental area, for which a corresponding item does not exist in the Anatomical Automatic Labeling, was created with a 5-mm radius sphere centered on the peak site, as described in a previous study^58^. A covariance analysis was performed with sex, age, sleepiness, and recognition of non-conscious images as covariates for voxels within the masks to compare the various conditions between the HC and PT groups. A small volume correction for the multiple comparisons was performed at the peak level within the masks. The risk level for the multiple comparison correction was set at a family-wise error of 5% at peak level.

### MRI partial correlation analysis: severity of insomnia and depression scale scores

The sites with significant differences in the non-conscious happy–neutral contrasts were extracted using MarsBaR, and their correlations with the AIS, PSQI, and SDS scores were studied. There were two clusters in the right insula; thus, the cluster with higher statistical values was used for analysis. Furthermore, SDS scores were significantly higher in the PT group. A partial correlation analysis was performed to show that the high SDS scores in the PT group were attributed to insomnia and not to a spurious correlation with high depression scores to assess whether the correlations with the PSQI and AIS were still significant after controlling for the severity of depression as measured using the SDS. Multiple comparison correction was performed by adjusting the false discovery rate: FDR (Benjamini and Hochberg^59^ method).

### Other statistical analyses

A t-test (two-tailed) was used to analyze the questionnaire scores and behavioral data under various conditions for the HC and PT groups. Correlations between inter-participant variables were analyzed by calculating Pearson’s product-moment correlation coefficient. SPSS PASW statistics 18 (International Business Machines Corporation, New York, USA) was used for the statistical analyses.

## Results

### Basic parameters of the participant groups

The data of each participant group are shown in Table 1. There were no significant differences in age, sex ratio, subjective sleepiness, recognition of the masked image, or button-pressing reaction time between the two groups. The PSQI, AIS, and SDS scores were significantly higher in the PT group than in the HC group.

**Table 1:** Participant demographic data.

	HC	PT	*t*	p
Age	60.5 (15)	60.3 (16.5)	0.6	ns
Sex ratio (M:F)	21:9	11:4	χ^2^ = 0.18	ns
PSQI	4 (1.76)	12.73 (2.4)	5.92	< 0.001
AIS	2.2 (1.73)	9.8 (2.98)	4.31	< 0.001
SDS	32.87 (7.33)	39.4 (6.41)	2.82	< 0.01
Sleepiness	0.51 (0.6)	0.93 (1.06)	0.91	ns
Awareness	1.33 (0.47)	1.53 (0.83)	0.87	ns
Number of responses	11.4 (2.07)	11.6 (1.17)	0.4	ns
Reaction time (ms)	390.4 (184.7)	436.3 (144.2)	0.97	ns

All values are expressed as mean (SD).

*HC* Healthy control group, *PT* Insomnia patient group, *PSQI* The Pittsburgh Sleep Quality Index, *AIS* Athens Insomnia Scale, *SDS* Self-Rating Depression Scale.

Degrees of freedom (df) = 43.

### Comparison of the fMRI data

To investigate the functional changes in the brains of the insomnia patients, we compared the activity in the regions of their brain responsible for emotions to that in healthy controls. The sites with significant differences and their statistical values are shown in Table 2.

**Table 2:** Brain regions with statistically significant differences in activation between insomniacs and healthy controls (p < 0.05, small volume correction).

Brain region			BA	MNI			*t*	Cluster k*
				x	y	z		
**Non-conscious fear minus neutral, HC minus PT**								
Left	Insula		13	− 38	− 18	14	4.78	40
Left	Insula		13	− 36	− 10	− 8	3.64	7
**Conscious happy minus neutral, HC minus PT**								
Right	Inferior orbitofrontal		47	42	36	− 6	3.91	15
**Non-conscious happy minus neutral, HC minus PT**								
Left	Lentiform nucleus	Caudate Head		− 4	10	0	5.57	361
		Putamen		− 16	12	− 4	4.39	
		Caudate body		− 10	− 2	14	4.32	
Right	Lentiform nucleus	Putamen		24	4	− 2	5.24	127
Right	Insula		13	38	14	6	4.52	37
Left	Inferior orbitofrontal		47	− 46	30	− 6	4.35	110
Right	Inferior orbitofrontal		47	42	40	− 6	4.09	39
Right	Insula		13	38	22	− 6	3.89	27
	Midbrain			2	− 20	− 16	2.99	(35^†^)

*BA* Brodmann area, *MNI* Montreal Neurological Institute template.

Cluster k*p < 0.001 threshold.

^†^p < 0.05 threshold (p-value for the ventral tegmental area cluster was greater than 0.001, but significant at peak level small volume correction); HC = healthy control group; PT = insomnia patient group.

There were no significant differences between the groups in brain activity in response to the conscious presentation of fear in any of the observed regions.

**Figure 2:**
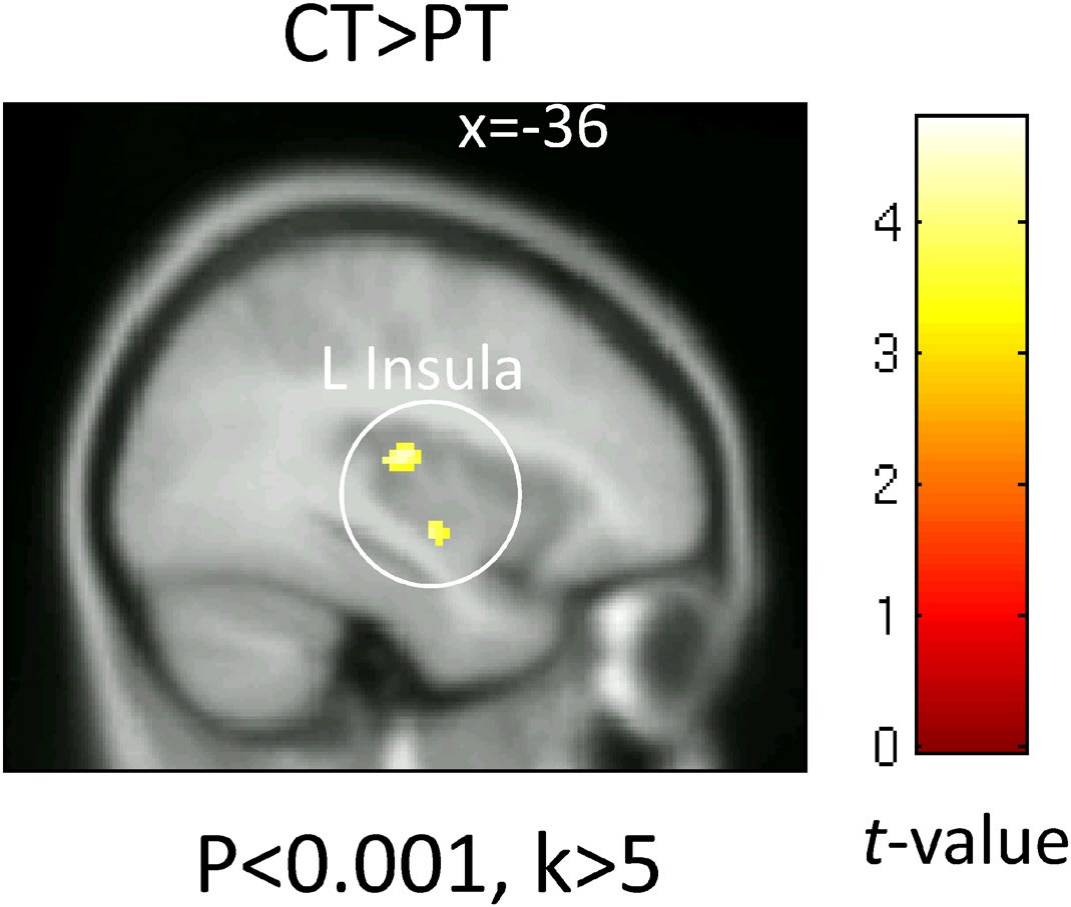
Brain activity in response to the non-conscious presentation of fear. The map shows a significantly greater activation in response to non-conscious fearful face stimuli in the healthy control than the insomnia patient group. Significant differences were observed in the left insula. Significant clusters are rendered on a T1 anatomical referential image displayed in neurological convention, with the left side corresponding to the left hemisphere.

Compared to the healthy controls, insomnia patients had significantly lower activity levels in the middle and posterior parts of the left insula in response to the non-conscious presentation of fear (Fig. 2: p < 0.05, small volume correction). There were no areas of the brain with more activity in the insomnia patients than in healthy controls.

**Figure 3:**
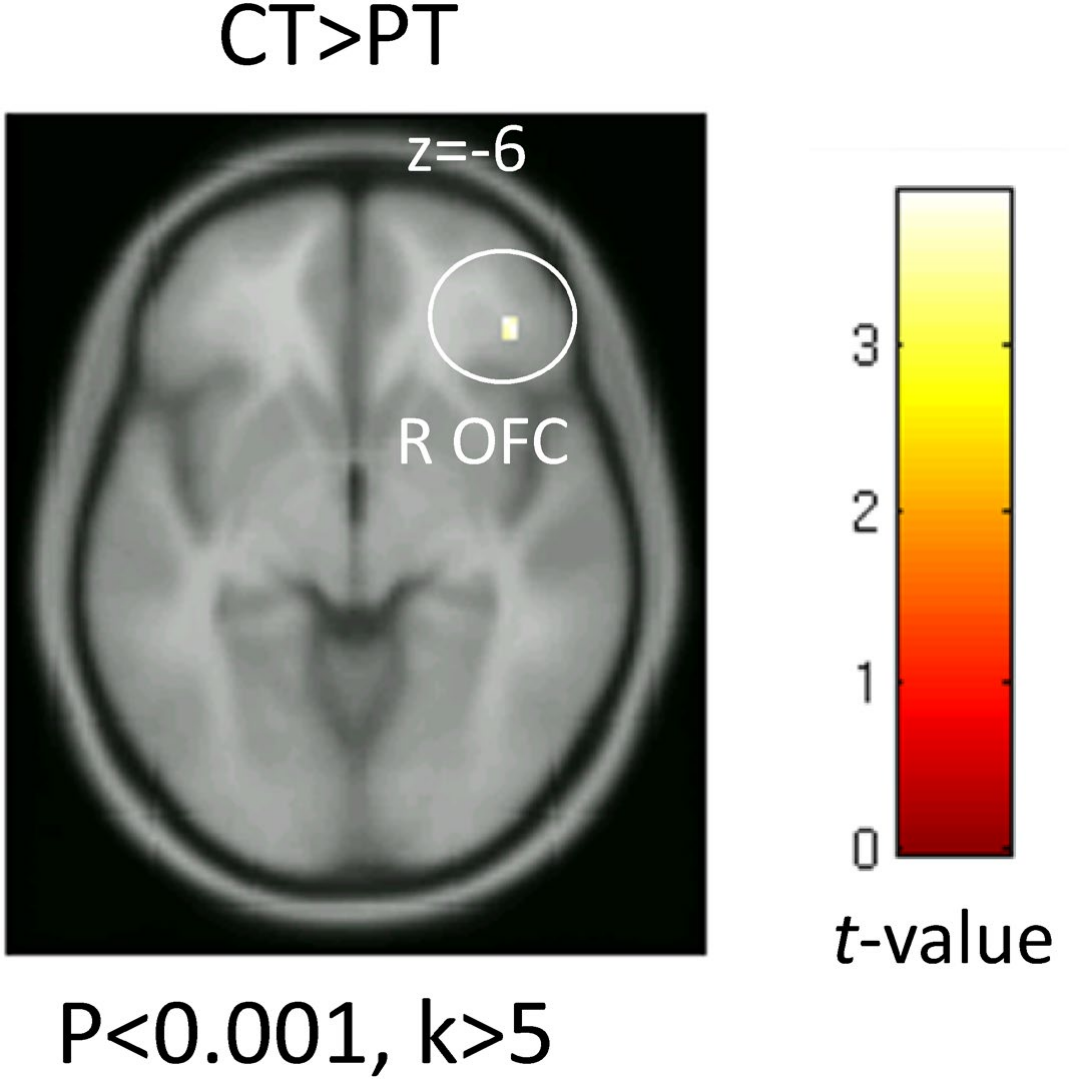
Brain activity in response to the conscious presentation of happiness. The map shows a significantly greater activation in response to conscious happy face stimuli in the healthy control than the insomnia patient group. Significant differences were observed in the right orbitofrontal cortex (OFC). Significant clusters are rendered on a T1 anatomical referential image displayed in neurological convention, with the left side corresponding to the left hemisphere.

The activity in response to the conscious presentation of happiness was significantly decreased in the right orbitofrontal cortex of insomnia patients compared to that of healthy controls (Fig. 3; p < 0.05, small volume correction). There were no significant differences in the brain activity in response to the facial expressions presented under other conditions. There were no sites showing a greater activity in the insomnia patients than in healthy controls.

**Figure 4:**
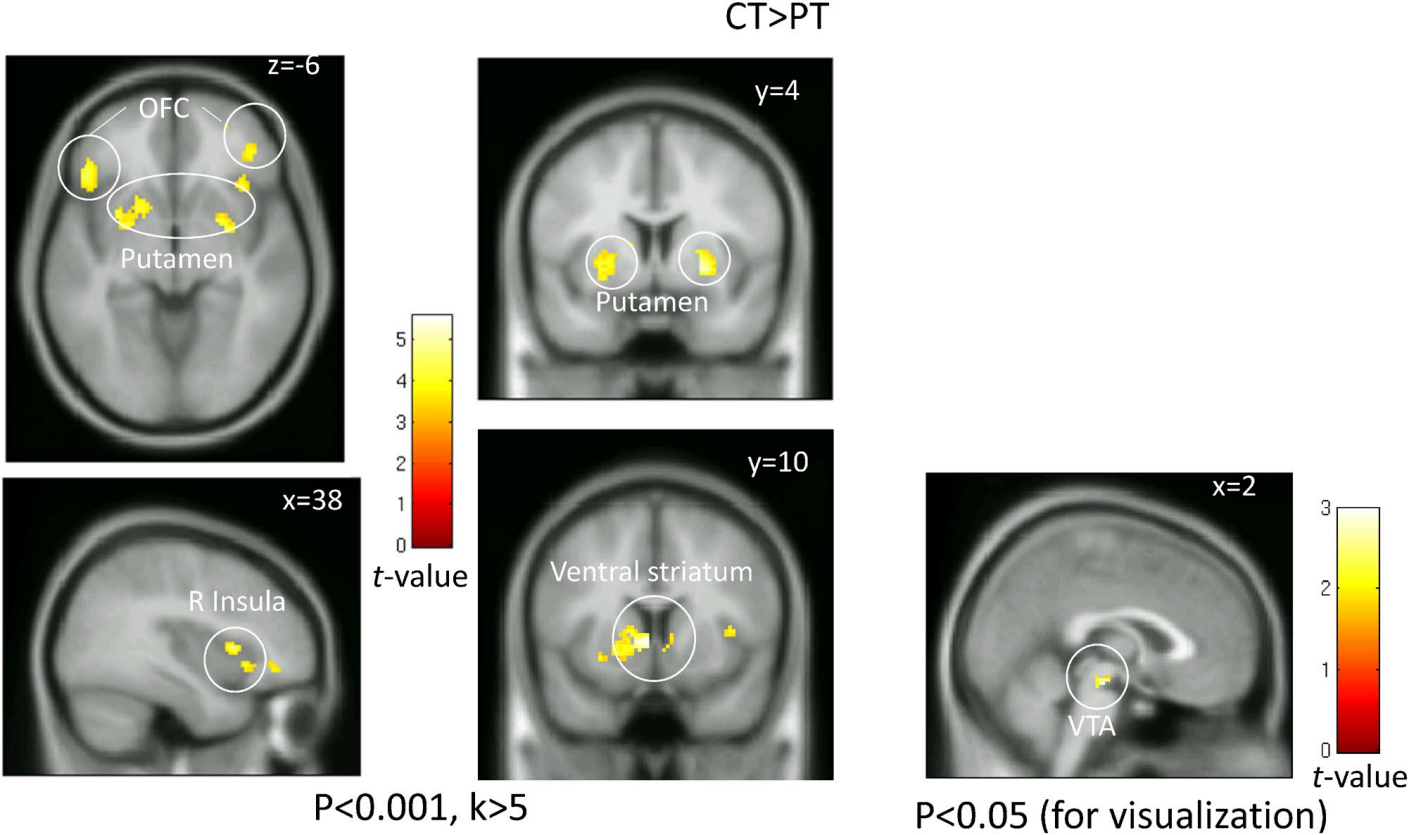
Brain activity in response to the non-conscious presentation of happiness. The map shows a significantly greater activation in response to non-conscious happy face stimuli in the healthy control than the insomnia patient group. Significant differences were observed in the ventral striatum, putamen, orbitofrontal cortex (OFC), right insula, and ventral tegmental area (VTA). Significant clusters are rendered on a T1 anatomical referential image displayed in neurological convention, with the left side corresponding to the left hemisphere. The cluster shown on the VTA were made using a threshold with a lenient alpha level (p < 0.05, k > 5) for visualization purposes.

In response to the non-conscious presentation of happiness, the activity in the ventral striatum, putamen, ventral tegmental area, bilateral orbitofrontal cortices, and the right anterior insula was significantly lower in insomnia patients than in healthy controls (Fig. 4: p < 0.05, small volume correction). There were no significant differences in the brain activity in these regions in response to the presentation of facial expressions in other conditions. There were no sites with greater activity in the insomnia patients than in healthy controls.

### Correlation analysis

The activity in the areas associated with reward had a significant negative correlation with the PSQI and AIS scores. The ventral tegmental area also showed similar correlations, though the correlation was not significant. SDS scores had a significant negative correlation with the activity in the right orbitofrontal cortex and ventral striatum, but not with that in the right insula (Table 3). All significant results survived after FDR correction.

**Table 3:** Correlation matrix analyses between the brain activity and scales of insomnia and depression.

	R insula	L OFC	R OFC	Putamen	VTA	vSTR
PSQI	− 0.438**	− 0.415**	− 0.492**	− 0.511**	− 0.432**	− 0.543**
AIS	− 0.485**	− 0.443**	− 0.503**	− 0.501**	− 0.284^†^	− 0.519**
SDS	− 0.273^†^	− 0.198	− 0.329*	− 0.225	0.021	− 0.313*

*L* left side, *R* right side, *OFC* orbitofrontal cortex, *VTA* ventral tegmental area, *vSTR* ventral striatum, *PSQI* The Pittsburgh Sleep Quality Index, *AIS* Athens Insomnia Scale, *SDS* Self-Rating Depression Scale.

^†^p < 0.1, *p < 0.05, **p < 0.01.

Degrees of freedom (df) = 45.

A partial correlation analysis with SDS scores as a control variable showed that the correlations between the brain activity and severity of insomnia were significant for all areas of the brain studied (Table 4). All significant results survived after FDR correction.

**Table 4:** Partial correlation matrix analysis after controlling for depression scale scores.

	R insula	L OFC	R OFC	Putamen	VTA	vSTR
PSQI	− 0.379*	− 0.376*	− 0.426**	− 0.473**	− 0.469**	− 0.487**
AIS	− 0.425**	− 0.404**	− 0.428**	− 0.459**	− 0.321*	− 0.452**

*L* left side, *R* right side, *OFC* orbitofrontal cortex, *VTA* ventral tegmental area, *vSTR* ventral striatum, *PSQI* The Pittsburgh Sleep Quality Index, *AIS* Athens Insomnia Scale.

*p < 0.05, **p < 0.01.

Degrees of freedom (df) = 42.

## Discussion

The present study revealed that the activity in the brain’s reward system (corpus striatum, ventral tegmental area, orbitofrontal cortex, ventral tegmental area, and right insula) in response to facial expressions of happiness were decreased in insomnia patients relative to that in controls. This is the first study to demonstrate that the changes in cerebral functions in response to positive emotions are altered in insomnia patients. Furthermore, we found that these patterns were observed after controlling for the symptoms of depression and that the severity of insomnia had a strong negative correlation with the activity in these brain regions.

The corpus striatum comprises of the putamen, caudate nucleus, nucleus accumbens, and other structures, and is well known to be associated with motivation and mood modulation^60,61^. The activity in the corpus striatum has been strongly associated with stimuli involving positive valence or reward, and this activity correlates strongly with the subjective assessment of valence^51–53^. The corpus striatum and other areas, such as the orbitofrontal area and insula, are also involved in goal-directed behaviors motivated by food or monetary rewards^54–57^. A meta-analysis of reward-related tasks^62^ has shown that the corpus striatum is associated with both the prediction and consumption of the reward. The corpus striatum receives dopaminergic projections from the ventral tegmental area of the midbrain and forms the neural networks responsible for the motivation, reinforcement, and reward processes with the surrounding areas, such as the insula, anterior cingulate cortex, and amygdala^63^. Activity in this dopaminergic system has been documented to play an important role in modulating the brain activity in response to positive rewards^60,61^.

The activation of the amygdala has been reported in several studies of non-conscious happy facial expression presentation tasks^64,65^. Many previous studies have reported that the presentation of non-conscious emotional facial expressions can elicit behavioral and physiological responses^66^, such as judgement modulation^67^, a modification of consumption behavior^68^, and non-consciously synchronized facial expressions^69^. The difference in the activity level between the amygdala and anterior cingulate cortex in response to the non-conscious presentation of happy faces versus sad faces has been reported to decrease with antidepressant therapy^70^. Though only a small number of studies have investigated the responses in the corpus striatum and insula, Chen et al. showed that the ventral striatal activity in response to the non-conscious (masked) presentation of an image of a close friend’s face smiling predicted subsequent developments in the friendship, and that it seemed to reflect the activity in the reward system^71^. The present study is the first to reveal that activity in the amygdala and ventral striatum in response to positive expression (happy) stimuli presented non-consciously is decreased in insomnia patients.

Only a few fMRI studies have investigated the reward processes in insomnia patients. One study assessed the brain activity in adolescent girls as they performed a monetarily rewarding task and detected no changes in the activity in the reward system in the brain, but found that the activity in the dorsomedial prefrontal cortex could be a mediator of the relationship between insomnia and depressed mood^72^. Wang et al. reported an altered corpus striatum resting-stage functional connectivity between the default mode network and the sensorimotor network in primary insomnia patients^73^. Patients with a history of bipolar disorder have decreased activity in the ventral striatum and orbitofrontal cortex in response to the conscious presentation of happy expressions compared to healthy individuals^74^. Decreased amygdala activity has been observed in patients with severe depression in response to a happy expression stimulus, but this returned to normal levels during remission^75^. Furthermore, activity in the amygdala and nucleus accumbens in response to happy expressions is lower in children and young adults at a high risk of depression^76^. Anhedonia in patients with depression correlates negatively with the amygdala and ventral striatum activity associated with the presentation of happy expressions^77^. These examples show that the activity of the neural networks related to emotions and rewards is decreased in individuals with, or at risk of, depression. The similarities between the reactions of the insomnia patients in these studies and those in the present study may be related to the lack of motivation and high depressive mood during the day in insomnia patients, and their higher risk of developing depression. Moreover, for the non-conscious presentation of happiness, the severity of insomnia predicted the activity of these regions, even after controlling for depression scores. This finding shows that the difference between insomnia patients and healthy individuals observed was not a spurious correlation attributed to the highly depressive state of insomnia patients, but supports the hypothesis that insomnia is directly linked to abnormal affective functions. Patients with chronic insomnia have been shown to exhibit an excessive craving for sleep and abnormal attention to sleep-related stimuli^78,79^. Dai et al.^80^ reported that the hyperactivity of the value-based attentional networks was associated with an excessive attention to sleep-related stimuli. Furthermore, an association between sleep craving and impaired function of the exploratory system, including the reward system, has been reported, and the association between these modulations of brain function and hyperarousal in chronic insomnia patients has been discussed. Moreover, the association between the exploratory system and the value-based attentional networks was shown to be reduced in insomnia patients than in healthy controls. Our findings of reduced activity in the reward system are also consistent with these findings; this effect may be related to the pathogenesis of hyperarousal in insomnia, mediated by the impairment of the proper allocation of reward-based attention in these patients, causing them to pay excess attention to sleep-related stimuli.

We expected to find overactivity of the emotion-related areas in insomnia patients due to hyperarousal; however, contrary to our expectations, there was no increased activation of the emotion-related areas in the PT group compared with the HC group. Conversely, we found reduced activity in the reward network, suggesting that the hyperarousal in insomnia is not associated with an overactive state of the entire brain. In this study, the middle and posterior parts of the left insula were significantly less activated by the non-conscious presentation of fearful faces in insomnia patients than in the control group. A meta-analysis on the functions of the insula^81^ has suggested that there is a strong relationship between affective tasks and the right insula, but associations with the middle part of the left insula have not been confirmed. However, significant activity in this region has been observed in studies on somatosensory and motor functions. The decreased activity in the middle left insula in response to negative stimuli is likely explained by the altered physiological functioning^8–10^ associated with the hyperarousal in insomnia patients.

A significant difference between the two groups in the brain activity in response to the presentation of fear was only observed when the presentation was non-conscious. Conscious presentation of happy expressions generated a between-group difference in the orbitofrontal cortex only, while a difference was observed in a broader area of the reward system when the presentation was non-conscious. There are two hypothesized reasons for these differences in brain activity. First, the non-conscious presentation may have generated simple responses from the neighboring systems that were unaltered by the visuo-cognitive or high-order functions of the prefrontal lobe. Indeed, a previous study reported that the magnitude of the amygdalar response to non-consciously presented emotional expressions was a strong predictor of personality traits and subjective sleepiness^47,82^. Etkin et al. investigated the strength of the correlation between the amygdalar activity and intensity of anxiety when participants consciously or non-consciously viewed an expression of fear and found significant correlations between the intensity of anxiety and amygdalar activity in response to a non-conscious presentation. They attributed the high correlation between the non-conscious processes and anxious traits to the notion that, unlike conscious processes, the non-conscious presentation is not affected by an inhibitive response to the presentation of an image^82^. The second predicted reason is the ceiling effect. While the difference between the two groups in their conscious responses to images were negligible, the non-conscious presentation of images may have provided the optimal stimulus intensity to observe a difference between the two groups.

This study has several limitations. First, data on the subjective assessments of the facial expression stimuli were not collected. Thus, it is unknown whether the stimuli induced pleasant emotions in the participants. Because the assessment of emotional stimuli has been reported to lower the activity in the brain regions responsible for emotions^83,84^, we did not ask participants to make subjective assessments while performing the task. However, the activity of the reward system decreased in response to non-conscious presentation; therefore, we assume it to be a system that is not directly related to subjective pleasure. This indicates that subjective assessments would not be relevant to the results of this study.

The second limitation is related to the sampling method. Although the patients who participated in this study encompassed a wide age range, the mean age was approximately 60 years; thus, our findings may not be directly comparable to those of previous studies that have used younger participants. A sample with a broader age range should be selected for further investigations.

Third, in this study, the PT group had 3-day washout period for hypnotics which was managed by the attending physician. It is possible that the patients in this group experienced symptoms of rebound insomnia. These symptoms might enhance the differences in brain activity between the two groups.

## Conclusion

The present study revealed a decreased responsiveness to positive emotional stimuli in regions associated with emotions and rewards in insomnia patients. This is the first report to show changes in cerebral functions related to positive emotions in insomnia patients, compared with healthy controls. A reduced responsiveness to positive stimuli is also observed in patients with depression or bipolar disorder and may be associated with the highly increased risk of depression in insomnia patients. Future studies should compare insomniacs to depression patients, as well as study whether similar patterns in responsiveness are also observed in people with non-clinical insomnia. Insomnia patients often show increased activity of the sympathetic nervous system^11–13^ and higher levels of physiological markers of stress such as cortisol^14–18^, thus the relationships between these markers and brain activity should be investigated as well.
